# Supplementary material for: Temporal order and precision of complex stress responses in individual bacteria
Source: Mol Syst Biol. 2019 Feb 14;15(2):e8470. doi: 10.15252/msb.20188470 (PMC6375286; doi:10.15252/msb.20188470)
Supplement: Supplementary file 1 — Expanded View Figures PDF [file MSB-15-e8470-s001.pdf]

Expanded View Figures

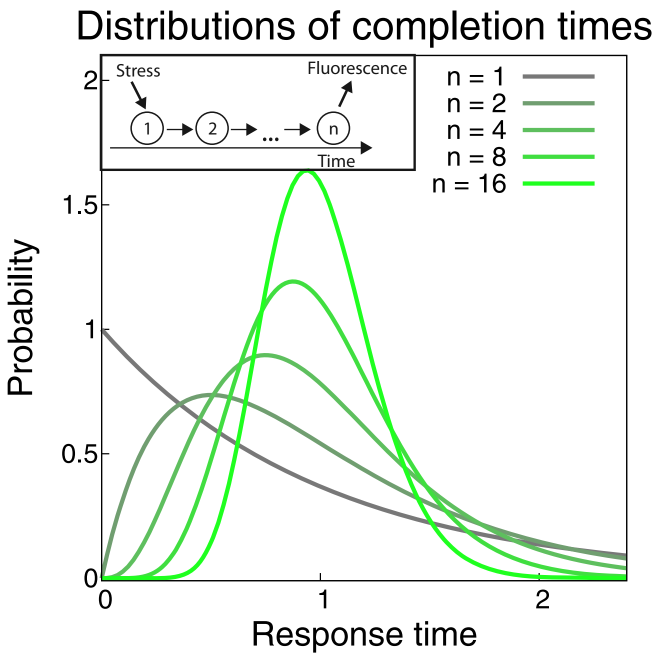

Figure EV1. Signal progression and the distribution of completion times.

Inset: A signal progresses through a system with *n* steps to elicit a measurable response after the addition of stress. The waiting time for each step is assumed to be exponentially distributed. Main panel: Erlang distributions of completion times for processes with different number of steps (increasing from gray to green), while keeping the mean completion time the same. More, but faster steps in the reaction lead to a tighter distribution around the mean, thus greater timing precision of the response.

Figure EV2. Growth rate fluctuations in representative microcolonies after TMP, TET, or NIT addition and their effect on timing variability determined with different response time measures.

- A Instantaneous growth rates (smoothed with moving average filter of window size 3) for each condition were determined for single cells (thin lines) in a microcolony, acquired with a microfluidics system (CellASIC). The thick lines represent the mean over all depicted single cells.
- B Same plot as in Fig 2C, but with the response time mean and standard deviations determined from the number of cell doublings instead of time. This analysis corrects for fluctuations in the growth rates of single cells. The dashed and dotted lines are the same as in Fig 2C.
- C Same data as in Fig 2A, but each cell was normalized to its maximum expression level (Materials and Methods). Response times in Fig EV2D–G were defined as the time until 50% of the maximum expression level was reached for each single cell.
- D Same plot as in Fig 2C, but with the response time determined as depicted in Fig EV2C.
- E Same plot as in Fig EV2B, but with the response time determined as depicted in Fig EV2C with cell doublings instead of time.
- F The randomness parameter $(\sigma_t/\mu_t)^2$ compared for the two tested response time measures. *X*-axis: Time until 25% of the median full expression of the microcolony was reached (see Fig 2A); *y*-axis: Time until half maximal expression was reached for each cell individually (see Fig EV2C).
- G Same plot as in Fig EV2F, but with the response times determined in units of the cell doubling time.
- H Standard deviation divided by the mean response time over the maximal absolute expression level (obtained from population-level measurements of the GFP-promoter library; Mitosch *et al*, 2017) for individual promoters after the addition of TMP, TET, or NIT. The Pearson correlation coefficient is -0.08 ± 0.23, error and *P*-value from bootstrapping (Materials and Methods).

▶

 © 2019 The Authors

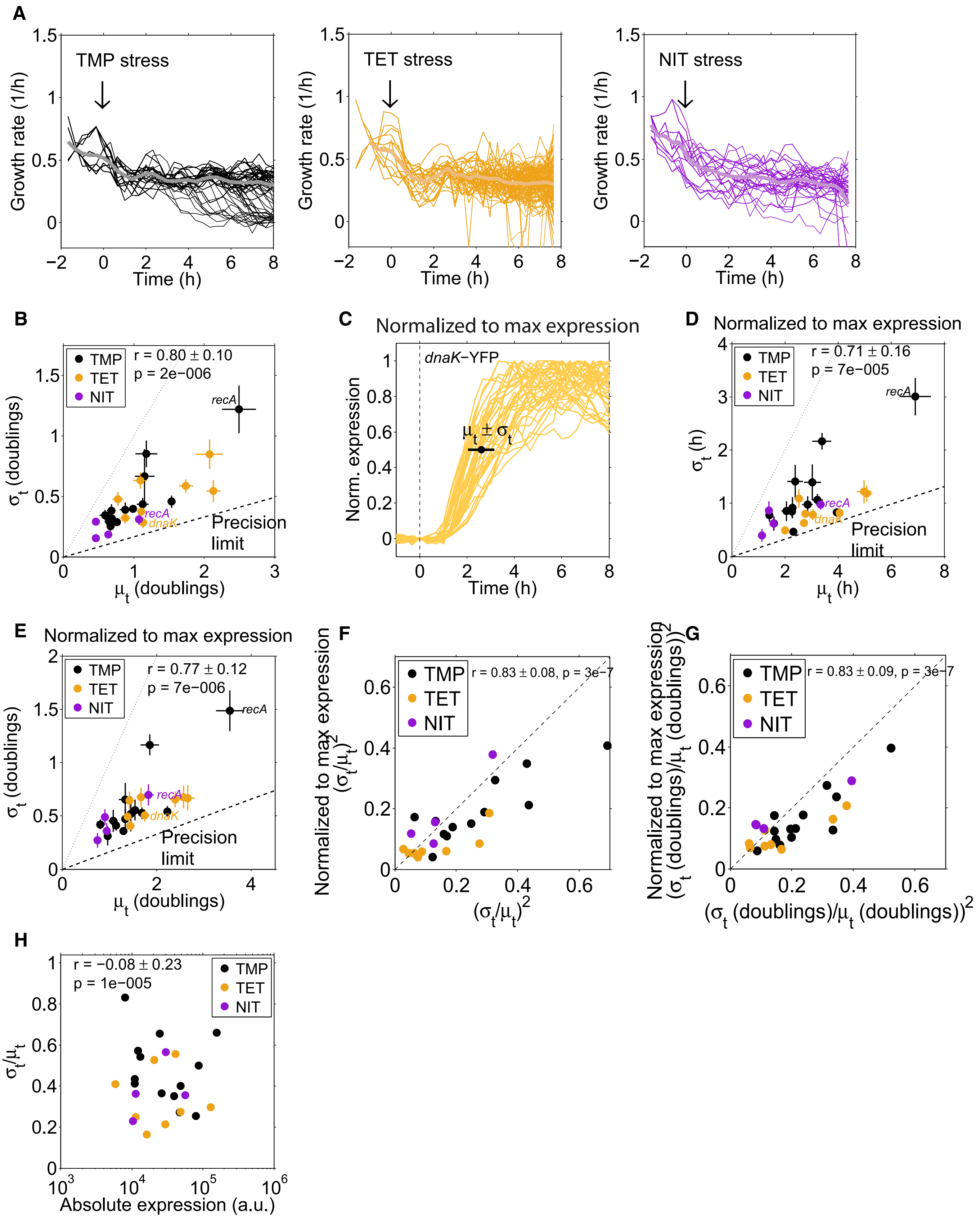
A
TMP stress
TET stress
NIT stress
Growth rate (1/h)
Time (h)
B
TMP
TET
NIT
r = 0.80 ± 0.10
p = 2e−006
recA
dnaK
Precision limit
σt (doublings)
μt (doublings)
C
Normalized to max expression
dnaK−YFP
μt ± σt
Norm. expression
Time (h)
D
Normalized to max expression
r = 0.71 ± 0.16
p = 7e−005
σt (h)
μt (h)
E
Normalized to max expression
r = 0.77 ± 0.12
p = 7e−006
F
r = 0.83 ± 0.08, p = 3e−7
Normalized to max expression (σt/μt)2
(σt/μt)2
G
r = 0.83 ± 0.09, p = 3e−7
Normalized to max expression (σt (doublings)/μt (doublings))2
(σt (doublings)/μt (doublings))2
H
r = −0.08 ± 0.23
p = 1e−005
σt/μt
Absolute expression (a.u.)

Figure EV2.

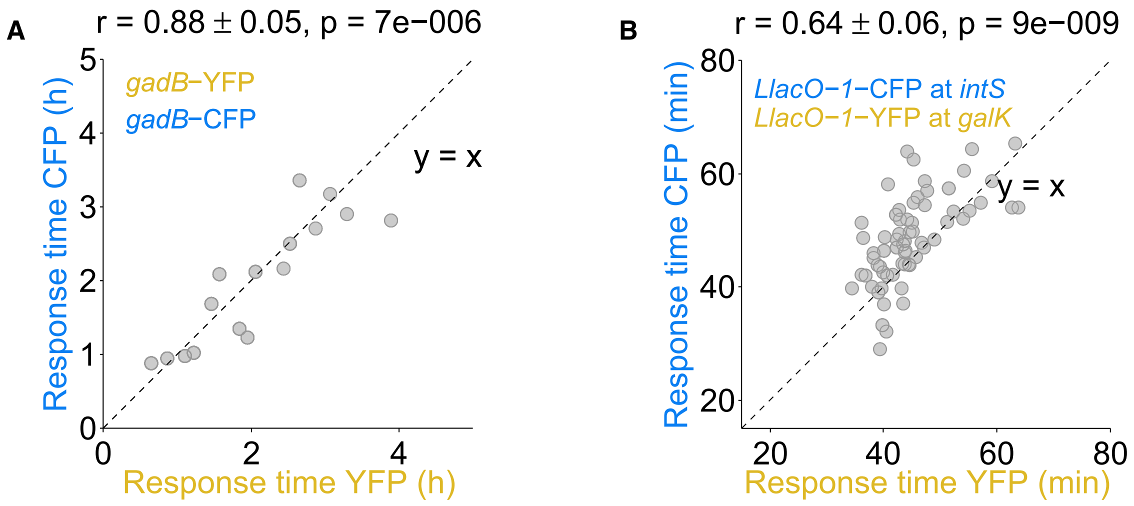

Figure EV3. Response time correlation between identical promoters.

A Response times for the acid stress promoter *gadB* combined with YFP at the *intS* locus and with CFP at the *galK* locus are highly correlated.

B Response times are also well correlated when swapping colors, i.e., combining the *LlacO-1* promoter with CFP at the *intS* locus and with YFP at the *galK* locus, respectively.

 © 2019 The Authors

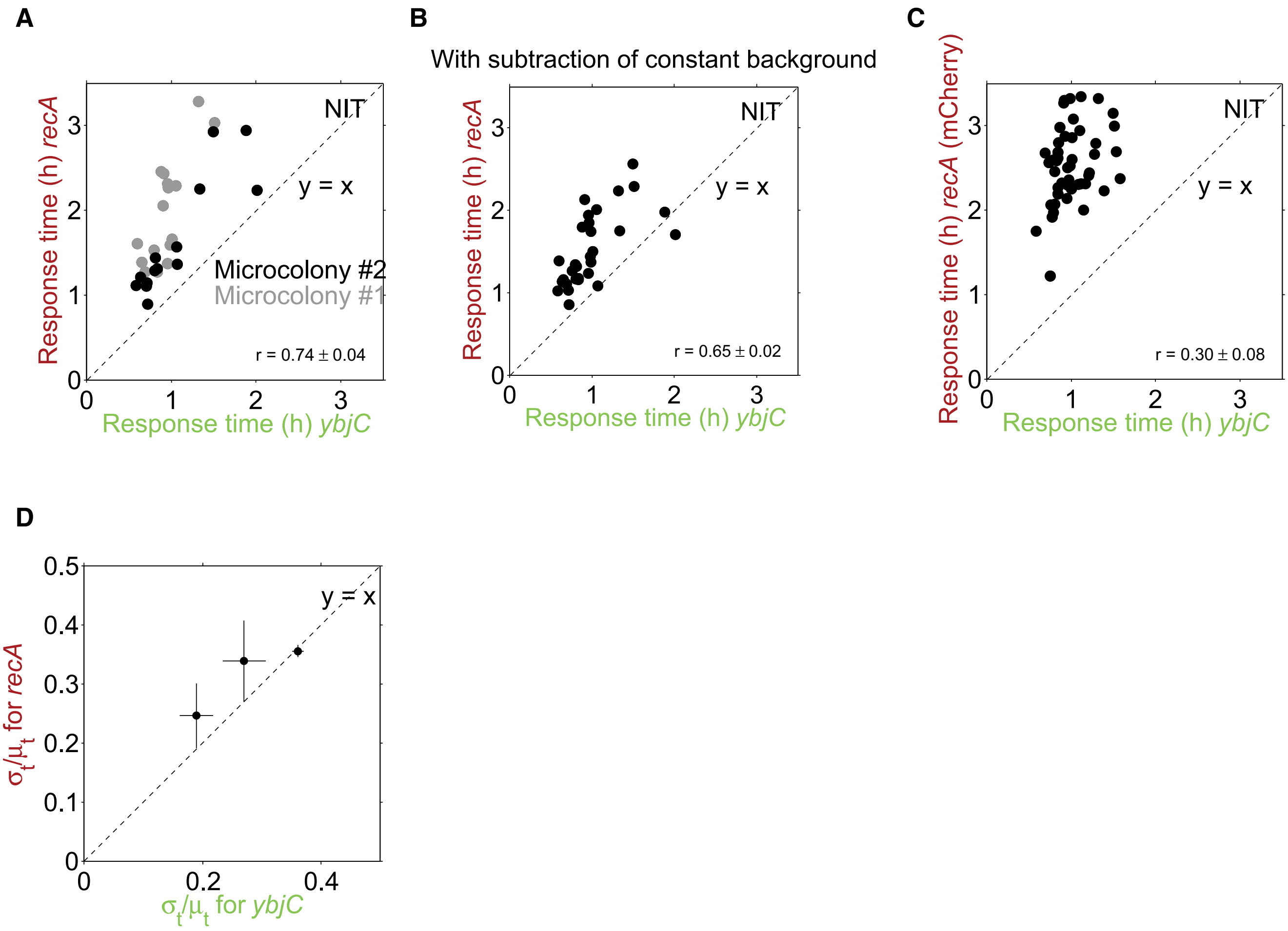

Figure EV4. Temporal order and response time correlation are independent of the specific microcolony, background subtraction, and the specific fluorophore used.

A Same data as in Fig 4C, but shown for each of the two microcolonies (black and gray data points, respectively). Clear temporal order and strong response time correlations are present in each microcolony.

B Same data as in Fig 4C, but background was subtracted as a constant for all time points and not specifically for each time point. Clear temporal order and response time correlation are independent of background subtraction ($r = 0.65$, $P = 9.9 \times 10^{-5}$).

C Response times for *ybjC*, combined as before with YFP, and *recA*, combined with mCherry instead of CFP as a control. The clear temporal order is also present here; the response time correlation is weaker ($r = 0.30$, $P = 0.05$). This may be explained by the longer maturation time of mCherry (Balleza *et al*, 2017) which may blur correlations.

D The ratio of σ_t/μ_t for *recA* versus *ybjC* from three replicate experiments. Error bars are from subsampling of descendants of single cells which were present at the time of stress addition (Materials and Methods).

Figure EV5. Gene expression and growth rate of knockout mutants under NIT stress, and temporal order for another oxidative stress promoter *fpr*.

- A The prodrug NIT is reduced to active nitro anion radicals by the nitroreductases NfsA and NfsB leading to oxidative stress. The oxidative stress transcription factor SoxS activates transcription of both nitroreductases (Keseler *et al*, 2017), potentially resulting in a positive feedback loop. The *nfsA* gene is in an operon with *ybjC*, controlled by the *ybjC* promoter.
- B Sequential temporal order and response time correlation between *ybjC* and *recA* are also present in a Δ*nfsA* strain.
- C Sequential temporal order is also present between another oxidative stress promoter, *fpr*, and *recA*. The positive response time correlation is absent.
- D Absorbance at 600 nm over time for the wild type and oxidative stress mutants (Δ*sodA*, Δ*gshA*, Δ*gshB*) under no stress condition and under 4 μg/ml NIT.
- E Same as (D), with 4 μg/ml NIT but the knockout strains were complemented with the respective gene (Materials and Methods), induced by 1 mM IPTG (Materials and Methods).
- F Expression of *ybjC*-YFP in response to 1.5 μg/ml NIT, added at $t = 0$, in the wild type and in a *gshA* knockout mutant. Lines are the mean, error bars are the standard deviation over the cells from three microcolonies for Δ*gshA* and two microcolonies for the wild type.
- G Expression of *recA*-CFP in response to 1.5 μg/ml NIT, added at $t = 0$, in the wild type and in a *gshA* knockout mutant. Lines are the mean, error bars are the standard deviation over the cells from three microcolonies for Δ*gshA* and two microcolonies for the wild type, the same microcolonies as in (F).

© 2019 The Authors

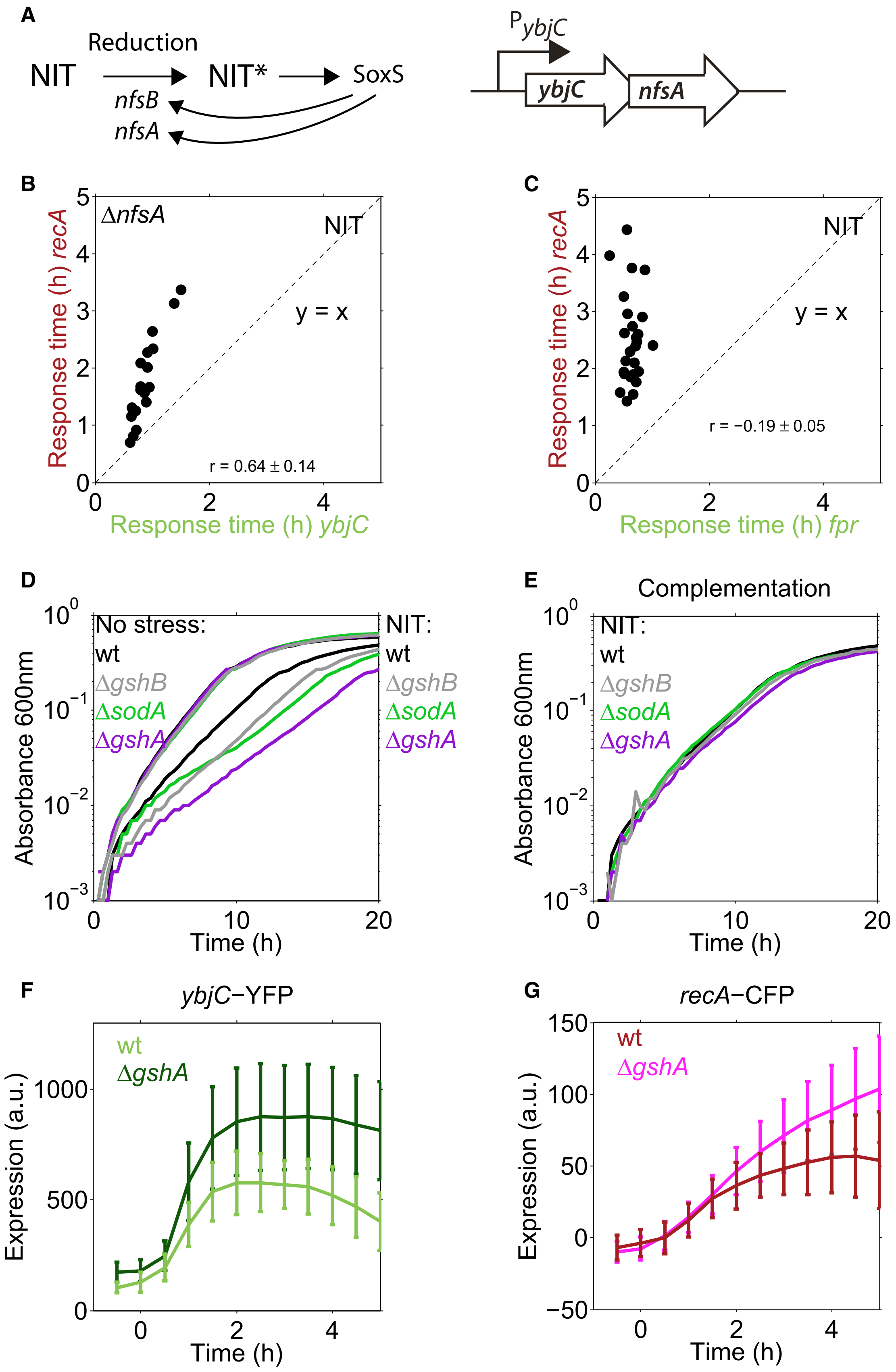
A
Reduction
NIT
NIT*
SoxS
nfsB
nfsA
PybjC
ybjC
nfsA
B
ΔnfsA
NIT
y = x
r = 0.64 ± 0.14
Response time (h) recA
Response time (h) ybjC
C
NIT
y = x
r = −0.19 ± 0.05
Response time (h) recA
Response time (h) fpr
D
No stress:
wt
ΔgshB
ΔsodA
ΔgshA
NIT:
wt
ΔgshB
ΔsodA
ΔgshA
Absorbance 600nm
Time (h)
E
Complementation
NIT:
wt
ΔgshB
ΔsodA
ΔgshA
Absorbance 600nm
Time (h)
F
ybjC−YFP
wt
ΔgshA
Expression (a.u.)
Time (h)
G
recA−CFP
wt
ΔgshA
Expression (a.u.)
Time (h)

Figure EV5.

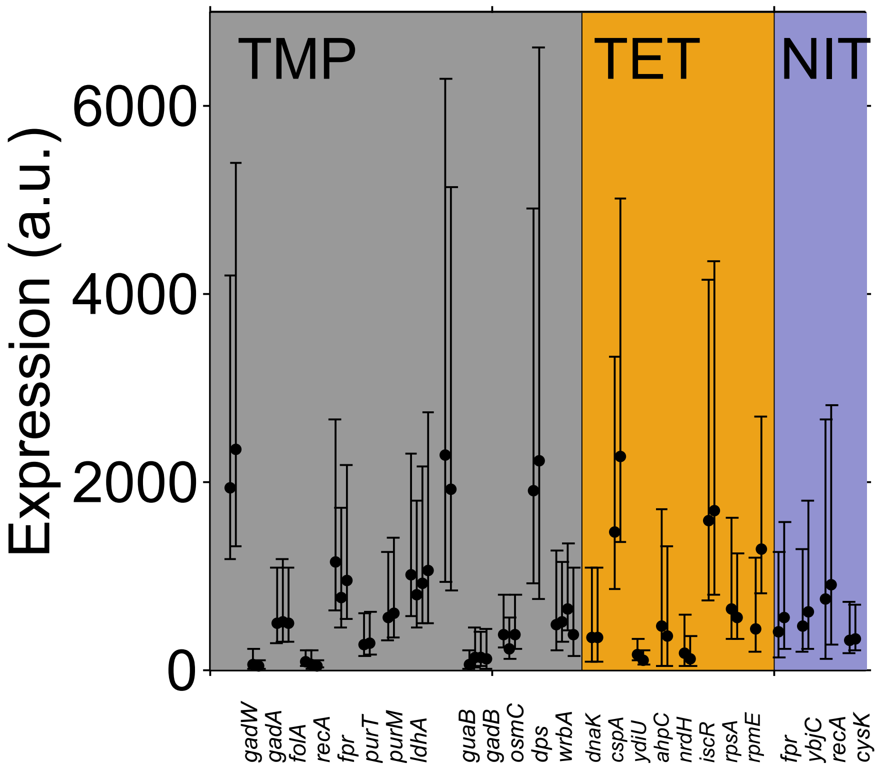

Figure EV6. Expression range and thresholds used for response time measurement in individual microcolonies under TMP, TET, or NIT stress for all studied promoters.

The lines depict the minimum and maximum median expression per microcolony; the dots depict the thresholds defined as 25% full median expression (see Materials and Methods). At least two microcolonies were analyzed per promoter and condition. Note that the expression levels are not directly comparable between different promoters due to different exposure times on the microscope. The two microcolonies for *rpmE* under TET were measured on different days and with different microscope settings.

 © 2019 The Authors
