# Supplementary material for: Temporal order and precision of complex stress responses in individual bacteria
Source: Mol Syst Biol. 2019 Feb 14;15(2):e8470. doi: 10.15252/msb.20188470 (PMC6375286; doi:10.15252/msb.20188470)
Supplement: Supplementary file 2 — Table EV1 [file MSB-15-e8470-s002.docx]

**Table EV1: Conditions and promoters depicted in Figure 2C and 2E.** Most promoters were measured with the YFP reporter at the *intS* locus, but some also with the CFP reporter at the *galK* locus (indicated with *).

| **Condition** | **Promoter-reporter** | **Chromosomal locus** | **Function** (Keseler *et al*, 2017) |
| --- | --- | --- | --- |
| TMP | *gadW-*YFP | *intS* | DNA-binding transcriptional dual regulator |
| TMP | *gadA-*YFP | *intS* | glutamate decarboxylase A |
| TMP | *folA-*YFP | *intS* | dihydrofolate reductase; gene product is the target of TMP |
| TMP | *recA-*YFP | *intS* | DNA recombination/repair protein RecA |
| TMP | *fpr-*YFP | *intS* | flavodoxin/ferredoxin-NADP^+^ reductase |
| TMP | *purT-*YFP | *intS* | phosphoribosylglycinamide formyltransferase 2 |
| TMP | *purM-*YFP | *intS* | phosphoribosylformylglycinamide cyclo-ligase |
| TMP | *ldhA-*YFP | *intS* | D-lactate dehydrogenase – fermentative |
| TMP | *guaB-*YFP | *intS* | IMP dehydrogenase |
| TMP | *gadB-*YFP | *intS* | glutamate decarboxylase B |
| TMP | *osmC-*YFP | *intS* | osmotically inducible peroxiredoxin |
| TMP | *dps-*YFP | *intS* | stationary phase nucleoid component that sequesters iron and protects DNA from damage |
| TMP | *wrbA-*YFP | *intS* | NAD(P)H:quinone oxidoreductase |
| TET | *dnaK-*YFP | *intS* | chaperone protein |
| TET | *cspA-*YFP | *intS* | DNA-binding transcriptional activator |
| TET | *ydiU-*YFP | *intS* | UPF0061 family protein |
| TET | *ahpC-*YFP | *intS* | alkyl hydroperoxide reductase, AhpC component |
| TET | *nrdH-*YFP | *intS* | glutaredoxin-like protein |
| TET | *iscR-*CFP* | *galK* | DNA-binding transcriptional dual regulator |
| TET | *rpsA-*YFP | *intS* | 30S ribosomal subunit protein S1 |
| TET | *rpmE-*YFP | *intS* | 50S ribosomal subunit protein L31 |
| NIT | *fpr-*YFP | *intS* | flavodoxin/ferredoxin-NADP^+^ reductase |
| NIT | *ybjC-*YFP | *intS* | DUF1418 domain-containing protein |
| NIT | *recA-*CFP* | *galK* | DNA recombination/repair protein RecA |
| NIT | *cysK-*YFP | *intS* | cysteine synthase A |
| IPTG | *LlacO-1-*YFP | *intS* | IPTG-inducible semi-synthetic promoter (Lutz & Bujard, 1997) |
