# Supplementary material for: Temporal order and precision of complex stress responses in individual bacteria
Source: Mol Syst Biol. 2019 Feb 14;15(2):e8470. doi: 10.15252/msb.20188470 (PMC6375286; doi:10.15252/msb.20188470)
Supplement: Supplementary file 3 — Table EV2 [file MSB-15-e8470-s003.docx]

**Table EV2: Response time correlation, number of observed cells n, and p-values for the correlation between *ybjC* and *recA* in triplicates, and for an additional control where CFP was exchanged by mCherry, measured on different days.**

| **Condition and promoters** | **Sample** | **Correlation ± standard deviation** | **p-value for correlation** | **n** |
| --- | --- | --- | --- | --- |
| NIT: *ybjC*-YFP and *recA*-CFP | Replicate 1 | 0.74 ± 0.04 | 3.8 · 10^-6^ | 30 |
| NIT: *ybjC*-YFP and *recA*-CFP | Replicate 2 | 0.46 ± 0.12 | 3.6 · 10^-2^ | 20 |
| NIT: *ybjC*-YFP and *recA*-CFP | Replicate 3 | 0.52 ± 0.07 | 2.8 · 10^-3^ | 33 |
| NIT: *ybjC*-YFP and *recA*-mCherry | Control | 0.30 ± 0.07 | 1.7 · 10^-2^ | 59 |
