# Supplementary material for: Temporal order and precision of complex stress responses in individual bacteria
Source: Mol Syst Biol. 2019 Feb 14;15(2):e8470. doi: 10.15252/msb.20188470 (PMC6375286; doi:10.15252/msb.20188470)
Supplement: Supplementary file 4 — Table EV3 [file MSB-15-e8470-s004.docx]

**Table EV3: Growth rates of non-responding cells (not crossing threshold) for respective promoters.**

| **Promoter** | **Mean of responding cells** | **Mean of non-responding cells** | **p-value** |
| --- | --- | --- | --- |
| *fpr* (TMP) | 0.32/h (n = 153) | 0.28/h (n = 27) | 0.03 |
| *recA* (TMP) | 0.33/h (n = 402) | 0.34/h (n = 18 ) | 0.57 |
| *gadW* (TMP) | 0.33/h (n = 115) | 0.35/h (n =38 ) | 0.15 |
| *gadA* (TMP) | 0.34/h (n = 317) | 0.33/h (n = 24) | 0.66 |
| *ldhA* (TMP) | 0.32/h (n = 93) | 0.37 (n = 4) | 0.12 |
| *osmC* (TMP) | 0.37/h (n = 146) | 0.31/h (n = 5) | 0.06 |
| *wrbA* (TMP) | 0.31/h (n = 155) | 0.25/h (n = 39) | 3.78 × 10^-6^ |
| *nrdH* (TET) | 0.33/h (n = 309) | 0.36/h (n = 35) | 7.40 × 10^-3^ |
| *recA* (NIT) | 0.36/h (n = 122) | 0.06/h (n = 1) | 0.09 |
