# Supplementary material for: Temporal order and precision of complex stress responses in individual bacteria
Source: Mol Syst Biol. 2019 Feb 14;15(2):e8470. doi: 10.15252/msb.20188470 (PMC6375286; doi:10.15252/msb.20188470)
Supplement: Supplementary file 5 — Table EV4 [file MSB-15-e8470-s005.docx]

**Table EV4. Primers used for the construction of chromosomally integrated promoter-FP strains.** Underlined bases are restriction sites.

| Primers used to exchange GFPmut2 for YFP:  cYFP-1: AGAAAGGATCCGAGAAGAACTTTTCACTGGAG and  cYFP-2: ATGACCTCGAGCTGAATGAACTGCAGGAC |
| --- |
| Primers used to exchange GFPmut2 for CFP:  cCFP-1: GTCCGGGATCCTCTAGATTTAAG and  cCFP-2: CTCGAGGGGATCCTCTAGATT |
| Primers used to integrate a CHL resistance into the plasmid p-KAN-YFP:  CmR-1: AGATACTCGAGGTGAAGACGAAAGGG and  CmR-2: AGAATCTCGAGTAGACGTCGATATCTGGCG |
| Primers used to integrate a KAN resistance into the plasmid p-AMP-CFP:  KanR-1: AGAATCTCGAGTCGGAATTGCCAGCTGGGGC and  KanR-2: ATGACAGGATCCTCGAACCCCAGAGTCCCGCTCAGAAG |
| Primers used for whole-plasmid PCR to make a defunct KAN resistance:  KanF: AGAATCTCGAGGATATCTGGCGAAAATGAGAC and CmR-1 |
| Primers used for whole-plasmid PCR to make defunct AMP resistance:  AmpF: ATGACCTCGAGAGTATTCAACATTTCCGTGT  CmEnd: CTAGTGCTTGGATTCTCACC |
| Primers used to integrate platform into *intS* locus:  intS-1: CCGTAGATTTACAGTTCGTCATGGTTCGCTTCAGATCGTTGACAGCCGCAGAGTCAGTGAGCGAGGAAGC and  intS-2:  ATAGTTGTTAAGGTCGCTCACTCCACCTTCTCATCAAGCCAGTCCGCCCATGAAGTCAGCCCCATACGAT |
| Primers used to integrate platform into *galK* locus:  galK-1: GTTTGCGCGCAGTCAGCGATATCCATTTTCGCGAATCCGGAGTGTAAGAAGAGTCAGTGAGCGAGGAAGC and  galK-2:  ACCATCGGGTGCCAGTGCGGGAGTTTCGTTCAGCACTGTCCTGCTCCTTGTGAAGTCAGCCCCATACGAT |
| Check primers for *intS* locus:  intS-up: GTACTTACCCCGCACTCCAT  intS-dn: TGTTCAGCACACCAATAGAGG |
| Check primers for *galK* locus:  galK-up: GTTAATTATCATTTTGCACCGCGTC and  galK-dn: GGAAAGTAAAGTCGCACCCC |
| Primers for the amplification of *Poi* from library plasmids for integration combined with YFP:  MKan-1: GCGATACCGTAAAGCACGAG and  mYFP: TTCTTCACCTTTGCTCATATGTATATCTCC |
| Primers for the amplification of *Poi* from library plasmids for integration combined with CFP:  AmpF2:  GACCAGGATAGGAACCACACCAGTAAACAGCTCCTCGCCCTTGCTCATATGTATATCTCCTTCTTAAATCTAGAG and  mCFP:  CAAAATGCCGCAAAAAAGGGAATAAGGGCGACACGGAAATGTTGAATACTCATACTCTTCCTTTTTCAATATTAT TGAAGCATTTATCAGGGTTATTGTCTCATGAGCGGATACATATTTGAAGACGTCTAAGAAACCATTATTATCATG |
| Primers to verify promoters integrated into platforms:  mKanProm_PromSeq: CAACCTTACCAGAGGGCG  mAmpProm_PromSeq: TTGTCTCATGAGCGGATACA |
| Primers to exchange the sequence for GFP by mCherry on the library plasmid:  mCh1: CCGCTCGAGAGATCCTCTAGATTTAAGAAGGAGATATACATATGGTTTCCAAGGGCGAGGAGG  mCh2: GCGCCTAGGTCTAGGGCGGCGGATTTGTCCTACTC |
| Primers to integrate the promoter-mCherry sequence into the CFP platform:  AmpF2:  GACCAGGATAGGAACCACACCAGTAAACAGCTCCTCGCCCTTGCTCATATGTATATCTCCTTCTTAAATCTAGAG  T1_XFP: TCTAGGGCGGCGGATTTGTCC |
