# Supplementary material for: Temporal order and precision of complex stress responses in individual bacteria
Source: Mol Syst Biol. 2019 Feb 14;15(2):e8470. doi: 10.15252/msb.20188470 (PMC6375286; doi:10.15252/msb.20188470)
Supplement: Supplementary file 6 — Table EV5 [file MSB-15-e8470-s006.docx]

**Table EV5. Promoter sequences of all promoters used in Figure 2C.** Underlined bases depict the start of the downstream gene. Promoters in which we detected single base pair mutations in the library plasmid (*gadA, gadB, fpr, ahpC, osmC*) were newly amplified from the MG1655 genome, cloned into the library plasmid and then integrated into the chromosome by lambda red-recombineering as described above.

| **Promoter** | **Sequence** |
| --- | --- |
| *gadW* | GATCGGCGCAGAGATTGTCGAACCGTGACTCGGCGGCAAGTATTGTTGCGCAAGGGAATTTTTACGGCACTGACCGTTCTGCGGAAGGAATAAGATTATAGAGTTTTACTCAGACATAAAAAAAACCCGGCATAGGGGACCGGGAAGAGGATAGTCTGCCGTCTCCAGACTAATAAACCGTTATAACACTCCCTGTTGGCACGGGAAACTTTGTGCTCTCAGTAAGTTAAATATAACTTTTACTGGAAATAAGATCAGCCATTTTTTTATAAACATAAGCTATACGCTGTGCGAAAATATATTCGTGCTGCATTTACTTATTATCAATTAACTGTTATGCAAAACTACTTTGTGGATAAATTTTGGTCCTACCAAATCTGGCAGTTTTTGCGCTAAGAAACAGTCTGGCATCATTTCATTAGTATACTGAAATTGAAATAATCGCAGTATGAAATATAAGGGATAATCATGACTCATGTCTGCTCGGTGATCCTCATTCGTCGTTCATTCGATATTTATCATGAACAGCAAAAAATATCGCTGCATAACGAGAGTATTCTGCTGCTGGAGAAAAATTTGGCAGACGATTTTGCGTTTTGTTCACCGGATACGCGACGACTGGATATCGATGAGCTGACAGTTTGCCATTACTTACAAAATATTCGTCAGCTACCACGCA |
| *gadA* | GCGGTGAAACTGATGCTGCGCTATCAGGTAGGCAAAGAGCTGCCGCAGGAGGATGTGGATGATATCGTAGCTTTCCTGCACAGTCTGAACGGGGTGTACACGCCGTATATGCAGGATAAACAATAATTAATTTGATCGCCCGAACAGCAATGTTTGGGCGATTTTTATTACGATAATAAAGTCTGTTTTTAATATTATCATGTTAAATGTTTATATTATAAAAAGTCGTTTTTCTGCTTAGGATTTTGTTATTTAAATTAAGCCTGTAATGCCTTGCTTCCATTGCGGATAAATCCTACTTTTTTATTGCCTTCAAATAAATTTAAGGAGTTCGAAATGGACCAGAAGCTGTTAACGGATTTCCGCTCAGAACTACTCGATTCACGTTTTGGCGCAAAGGCCATTTCTACTATCGCGGAGTCAAAACGATTTCCGCTGCACGAAATGCGCGATGATGTCGCATTTCAGATTATCAATGATGAATTATATCTTGATGGCAACGCTCGTCAGAACCTGGCCACTTTCTGCCAGACCTGGGACGACGAAAACGTCCATAAATTGATGGATTTGTCGATCAATAAAAACTGGATCGACAAAGAAGAATATCCGCAATCCGCAGCCATCGACCTGCGT |
| *folA* | GCAGGGAACCGAAGAAGGTAAACATACCGGCAACATGGCGGATGAACCGGAAACGAAACCCTCATCCTAATAAAGAGTGACGTAAATCACACTTTACAGCTAACTGTTTGTTTTTGTTTCATTGTAATGCGGCGAGTCCAGGGAGAGAGCGTGGACTCGCCAGCAGAATATAAAATTTTCCTCAACATCATCCTCGCACCAGTCGACGACGGTTTACGCTTTACGTATAGTGGCGACAATTTTTTTTATCGGGAAATCTCAATGATCAGTCTGATTGCGGCGTTAGCGGTAGATCGCGTTATCGGCATGGAAAACGCCATGCCGTGGAACCTGCCTGCCGATCTCGCCTGGTTTA |
| *recA* | TTGCAGACCTTGTGGCAACAATTTCTACAAAACACTTGATACTGTATGAGCATACAGTATAATTGCTTCAACAGAACATATTGACTATCCGGTATTACCCGGCATGACAGGAGTAAAAATGGCTATCGACGAAAACAAACAGAAAGCGTTGGCGGCAGCACTGGGCCAGATTGAGAAACAATTTGGTAAAGGCTCCATCATGC |
| *fpr* | CAAGTCACGCACCATTCGCCGCATTCAGTCCATCCACTATCTGGATCGCAAAGACCCGGAAATGCAGGTGCACATCCTCTGATTGATTTGATCGATTGAGCCTTCCAGTCCTTCGGGACTGGAATTTTTTTGTTCGGAGAACGAAGATAAGGCAAGTCAATCAAAACAGGAGAAAAACATGGCTGATTGGGTAACAGGCAAAGTCACTAAAGTGCAGAACTGGACCGACGCCCTGTTTAGTCTCACCGTTCACGCC |
| *purT* | TCGGCTTCCTTTTTGCTGGTAAACGACATTTTCTCTTCGCCCTCACGAATGACTACGTATTTAACTTCAACCGCCATTTGCAGCCTCTCATAATAACTGTGATTTTATACAGTATATTTCTTTTCGGTTGAGAAATCAACATCAGCAATAAAGACACACGCAAACGTTTTCGTTTATACTGCGCGCGGAATTAATCAGGGGATATTCGTTATGACGTTATTAGGCACTGCGCTGCGTCCGGCAGCAACTCGCGTGATGTTATTAGGCTCCGGTGAACTGG |
| *purM* | TTGCTCACGCATCAGTCCCAGCTTGTGTTTGACGAGTGGGTGTTTGACTTCCACGATCTTCATACTCTTTCTCCTTTGAGGGGCAGCCACAAAAAAAATCGACGGATTATACCTCCTTTCTTCAAGGCGGCAATATTCTTTTCGTTGACTTTAGTCAAAATGATAACGGTTTGAGATAAAGTTATTTTATATTCAGATGGTTATGAAAGAAGATTATTCCATCCGAAAACTAACCTTTACCCTGGCACAAGTCTTCTTTCGCCGCGCGCCTGGGGAAAAGACGTGCAAAAAGGTTGTGTAAAGCAGTCTCGCAAACGTTTGCTTTCCCTGTTAGAATTGCGCCGAATTTTATTTTTCTACCGCAAGTAACGCGTGGGGACCCAAGCAGTGACCGATAAAACCTCTCTTAGCTACAAAGATGCCGGTGTTGATATTGACGCGGGTAATGCTCTGGTT |
| *ldhA* | CGTCATCAGCAGCGTCAACGGCACAAGAATAATCAGTAATAACAGCGCGAGAACGGCTTTATATTTACCCAGCATGGGTAGTTAATATCCTGATTTAGCGAAAAATTAAGCATTCAATACGGGTATTGTGGCATGTTTAACCGTTCAGTTGAAGGTTGCGCCTACACTAAGCATAGTTGTTGATGAATTTTTCAATATCGCCATAGCTTTCAATTAAATTTGAAATTTTGTAAAATATTTTTAGTAGCTTAAATGTGATTCAACATCACTGGAGAAAGTCTTATGAAACTCGCCGTTTATAGCACAAAACAGTACGACAAGAAGTACCTGCAACAGGTGAACGAGTCCTTTGGCTTTGAGCTGG |
| *guaB* | CCGCTGATCCAAACCTGTCCCATCTCATGCTCAAGCAGCAGACGAACCGTTTGATTCAGGCGACTAACGGTAAAAATTGCAGGGGATTGAGAAGGTAACATGTGAGCGAGATCAAATTCTAAATCAGCAGGTTATTCAGTCGATAGTAACCCGCCCTTCGGGGATAGCAAGCATTTTTTGCAAAAAGGGGTAGATGCAATCGGTTACGCTCTGTATAATGCCGCGGCAATATTTATTAACCACTCTGGTCGAGATATTGCCCATGCTACGTATCGCTAAAGAAGCTCTGACGTTTGACGACGTTCTCCTCGTTCCTGCTCACTCTACCGTTCTGCCGA |
| *gadB* | TCCTGCAGCATGGACTGAGCAGGAGCAATTGTTGAAACAAATGACGGTAGAGAATGTTAACACTGCCGTTAAACAATATCTTTCTCATCCGGTAAATACTTATACCGGAGTATTATTGCCAAAATAATAACAGCCCCGTCAACACATCGTTGGCGGGGATTTTAGCAATATTCGCTATTTTTATGTAATAATTTTATAAATGCGTTCAAAATAATAATCAAGTACTAATAGTGATATTTTAAGGTCTGATTTTTACGTGATAATTCAGGAGACACAGAATGCGCATAAAAATAACAGCATAAAACACCTTACCACCACCCAAGAATTTCATATTGTATTGTTTTTCAATGAAAAAATATTATTCGCGTAATATCTCACGATAAATAACATTAGGATTTTGTTATTTAAACACGAGTCCTTTGCACTTGCTTACTTTATCGATAAATCCTACTTTTTTAATGCGATCCAATCATTTTAAGGAGTTTAAAATGGATAAGAAGCAAGTAACGGATTTAAGGTCGGAACTACTCGATTCACGTTTTGGTGCGAAGTCTATTTCCACTATCGCAGAATCAAAACGTTTTCCGCTGCACGAAATGCGCGACGATGTCGCATTCCAGATTATCAATGACGAATTATATCTTGATGGCAACGCTCGTCAGAACCTGGCCACTTTCTGCCAGACCTGGGACGACGAAAATG |
| *osmC* | TGCTGTTGAATTTTCTGCCTGATAATAAGTAAACATAGTGATTCTCCGTGTCTGTGTATTTATGGTGTCTGCTACGGATCGCAGATTTATAAAGCACATTCAGCATGGCAAATATTTGCCGCTTCGTTGTTAAGATTAGTCCTGGTTGATGATTTTTATATTTTAACACCATGATATTCATAGGGATTGTGATTGGTATGATCCGATTAATATTGATACAATATCTTTTGGGTTATATATTCCCGGTAATCTATTGTGGGAATTTAATTTAAGTGCAGAAGTAATATTTTCGCCGGATTTTATTCGGAATATCCTGCTTATCCTCGTGCTGTTTCTCACGTAGTCTATAATTTCCTTTTTAAGCCCACAGGAGAGCAACAATGACAATCCATAAGAAAGGTCAGGCACACTGGGAAGGCGATATCAAACGCGGGAAGGGAACAGTATCCACCGAGAGTGGCGTGCTGAACC |
| *dps* | CCGCTTCAATGGGGTCTACGCTGACAGTACGCAAAGAGAGCAAAATAAAAGAATTAGACATTAATTAAATTTACATTTCTGCATGGTTATGCATAACCATGCAGAATTTCTCGCTACTTTTCCTCTACACCGTCTTTATATATCGAATTATGCAAAAGCATATTTATTCCGAAAATTCCTGGCGAGCAGATAAATAAGAATTGTTCTTATCAATATATCTAACTCATTGAATCTTTATTAGTTTTGTTTTTCACGCTTGTTACCACTATTAGTGTGATAGGAACAGCCAGAATAGCGGAACACATAGCCGGTGCTATACTTAATCTCGTTAATTACTGGGACATAACATCAAGAGGATATGAAATTATGAGTACCGCTAAATTAGTTAAATCAAAAGCGACCAATCTGCTTTATACCCGCAACGATGTCTCCGACA |
| *wrbA* | TAGTGAACTGACGCGGGCAGACCCGCGTTTGGTAATATCCTGCAACAGCTTCTTAGCGAGAATAGATATCATCTCCAATAATTATCCATAAGCCGCTCTTATGATTAAAAAAACGCGATGAGATAAAAATGTGTCATTTTGCGACAAAATTACGTGCTTGTGAAAGTTGTTATAAATCAAATAAGTGGTTGTGAAATTTGCACTCTGAAAAGGACGTCTTATCTTTAAATAAGTGGTAGCGAATCGCTACGGAATAGAGATAACACGAGGAGTGGTTAGAAATGGCTAAAGTTCTGGTGCTTTATTATTCCATGTACGGACATATTGAAACGATGGCACGCGCAGTCGCTGAGGGTGCAAGCAAAGTGGATGGCGCTGAA |
| *dnaK* | TTTATGGTCGTTTGCCTGCGCCGTGCAGCACAGCATCAGGCTAATCGCCAGGCTGGCGGAAATCGTAAAAACGGATTTCATAAGGATTCTCTTAGTGGGAAGAGGTAGGGGGATGAATACCCACTAGTTTACTGCTGATAAAGAGAAGATTCAGGCACGTAATCTTTTCTTTTTATTACAATTTTTTGATGAATGCCTTGGCTGCGATTCATTCTTTATATGAATAAAATTGCTGTCAATTTTACGTCTTGTCCTGCCATATCGCGAAATTTCTGCGCAAAAGCACAAAAAATTTTTGCATCTCCCCCTTGATGACGTGGTTTACGACCCCATTTAGTAGTCAACCGCAGTGAGTGAGTCTGCAAAAAAATGAAATTGGGCAGTTGAAACCAGACGTTTCGCCCCTATTACAGACTCACAACCACATGATGACCGAATATATAGTGGAGACGTTTAGATGGGTAAAATAATTGGTATCGACCTGGGTACTACCAACTCTTGTGTAGCGATTATGGATGGCACC |
| *cspA* | AATCCAGACGCGTGAAGCCTTCAAGTGCCGAACTAAAATTGATGCGTTTGATTCAAGCCAACCCGGCATTAAGTAAGCAGTTGATGGAATAGACTTTTATCCACTTTATTGCTGTTTACGGTCCTGATGACAGGACCGTTTTCCAACCGATTAATCATAAATATGAAAAATAATTGTTGCATCACCCGCCAATGCGTGGCTTAATGCACATCAACGGTTTGACGTACAGACCATTAAAGCAGTGTAGTAAGGCAAGTCCCTTCAAGAGTTATCGTTGATACCCCTCGTAGTGCACATTCCTTTAACGCTTCAAAATCTGTAAAGCACGCCATATCGCCGAAAGGCACACTTAATTATTAAAGGTAATACACTATGTCCGGTAAAATGACTGGTATCGTAAAATGGTTCAACGCTGACAAAGGCTTCGGCTTCATCACTCCTGACGATGGCTCT |
| *ydiU* | TTTCCATGCTCTCCAGGGATGCCTGTGGCCTGCCGTTCCCATAAATCAGATAACGACGTTGGTTCAGCGATAACCCTTCTGTTTGCTGGTGTTTAAGACGAGAGTAACCGTCTACACTATCAAACAGGAGGATCTATGACCCTGTCTTTTGTTACCCGCTGGCGCGATGAATTGCCAGAAACCTATACAGCACTTTCCCCTACGCC |
| *ahpC* | GCAGGAAGCAGAGCCAGTAAAAGTATCTTTTTTAACATTAATTTGTCCTTTTCAGTCAGTGCAAAAGTCGAGTAAAAGGCATAACCTATCACTGTCATAGGTAAGAGCTTAGATCAGGTGATTGCCCTTTGTTTATGAGGGTGTTGTAATCCATGTCGTTGTTGCATTTGTAAGGGCAACACCTCAGCCTGCAGGCAGGCACTGAAGATACCAAAGGGTAGTTCAGATTACACGGTCACCTGGAAAGGGGGCCATTTTACTTTTTATCGCCGCTGGCGGTGCAAAGTTCACAAAGTTGTCTTACGAAGGTTGTAAGGTAAAACTTATCGATTTGATAATGGAAACGCATTAGCCGAATCGGCAAAAATTGGTTACCTTACATCTCATCGAAAACACGGAGGAAGTATAGATGTCCTTGATTAACACCAAAATTAAACCTTTTAAAAACCAGGCATTCAAAAACGG |
| *nrdH* | GACCCTGGTGTAGCGTGGGTACAGCAGCTGCGGTAGGTATTTTTATCGGCGCACTGTTAAGCATGCGCAAATCGTAGTGCAAAAATGATAATAAATACGCGTCTTTGACCCCGAAGCCTGTCTTCGGGGTTTCTTTTTGCCTGGTGAATCACAAAAATCCCCCTACCCCGTCACGCTCATATCCAGGGTAATTTCGACCACTATTTGCTATATATTGTGTGGTTGAATCTTTTTTCAACTACATCTAGTATCTCTGTATCAACAGAGAGACAACCCGACGCGTATCATCGCGCCGTATCTTCATTTTAAACGGAAATACGAATCATGCGCATTACTATTTACACTCGTAACGATTGCGTTCAGTGCCACGCCACCAAACGGGCGATGGAAAACCGGGGCTTTGATTTTGAA |
| *iscR* | CGGAAAGCCAGGAGTTGAATATCCTGCGCGGGATTCTGGCTTCTATTGAGCAGCAGAATAAAGGTAACAAGGCCGAATAACAGCCGTTGCCTGATGCGACGCGTAATGCGTCTTATCAGGCCTACAGTGAACAGAACCGTAGGTCGGATAAGGCGTTCACGCCGCATCCGACAGCCGTTGCCTGATGCGACGCGTAATGCGTCTTATCAGGCCTACAGTGAACAGAACCGTAGGTCGGATAAGGCGTTCACGCCGCATCCGACAGCCGTTGCCTGATGCGACGCGTAATGCGTCTTATCAGGCCTACAGTGTACAGAACCCCAGGGCGGATATGGCGTTCACGCCGCATCCGACAACAGGTACAAACGCCACGATAAAAAAATGGCACTGAAGGTTAAATACCCGACTAAATCAGTCAAGTAAATAGTTGACCAATTTACTCGGGAATGTCAGACTTGACCCTGCTATGCAATACCCCCACTTTTACAATAAAAAACCCCGGGCAGGGGCGAGTTTGAGGTGAAGTAAGACATGAGACTGACATCTAAAGGGCGCTATGCCGTGACCGCAATGCTTGACGTTGCGCTCAACTCTGAAGCGGGC |
| *rpsA* | GGCAGCCGATGCTTTAGTGTTGGATTCCACCACCTTAAGCATTGAGCAAGTGATTGAAAAAGCGCTACAATACGCGCGCCAGAAATTGGCTCTCGCATAAGCGACCGAATTTGCAGTACCCCCGTTGCAATGGAATGACAGCGGGTATGTTAAACAACCCCATCCGGCATGGAGCCAGGTGGACGTTAAATATAAACCTGAAGATTAAACATGACTGAATCTTTTGCTCAACTCTTTGAAGAGTCCTTAAAAGAAATCGAAACCCGCCCGGGTTCTATCGTTCGTGGCGTTGTT |
| *rpmE* | AACCGTCATGCCTTCTGGCAGCAGATAGTCAAAGGTACGAGGAAGCGGAACGGGCAAGGCAACGTGGGCAACGGGCATAGCATCATCCTGACTTGAAATTCGGTGGGTTAGTATACACATTGCCGTAGAAGAGTGCGGATCAGTTTGCATACGCTGGTTAATTTCTGTATGATTTCGCGCCTTCGTACGAAATGATCGTATTGAAGCTATACTTTTAACATCGCGTGGTGTCTGGCGTTAGGGCTGGAAGAGCGACGCGGCCTTAAACCGAGGTTTTCCCATGAAAAAAGATATTCACCCGAAATACGAAGAAATTACTGCTAGCTGCTCTTGCGGTAACGTAATGAAAATCCGCTCCACCGTT |
| *ybjC* | CATCGCGTTCATTGCTCAATTTCTCAGCCAGATCTTTTGCACGCACACAGTAAGGGCAACCCGAACGACCAAAAATAACGGTTTGCATTATTTCTCTCCTCATAGATTTATGCCTGTAATGATCACGCTAAAATGTATTCGCTGAAAGTAGGTTTAACCTGTTGCATTAATTGCTAAAAGCTATAACTGTTAAACACAATACAGTGAAAAGTTTTAGACTGAAGGCTCACTTTGCAGAGGGAAGCGTATGCGCGCGATCGGTAAATTGCCTAAAGGCGTGTTGATACTGGAATTTATCGGAATGATGCTACTGGCGGTGGCGCTGCTGTCGGTAAGCGACTC |
| *cysK* | TGTTCATCATGCCCGTTGCCGTTTGTGGCGCGACGGCGATGTGGGTCGATTGCTATCGCGATAAACACGCGATGTGGCGGTAACAATCTACCGGTTATTTTGTAAACCGTTTGTGTGAAACAGGGGTGGCTTATGCCGCCCCTTATTCCATCTTGCATGTCATTATTTCCCTTCTGTATATAGATATGCTAAATCCTTACTTCCGCATATTCTCTGAGCGGGTATGCTACCTGTTGTATCCCAATTTCATACAGTTAAGGACAGGCCATGAGTAAGATTTTTGAAGATAACTCGCTGACTATCGGTCACACGCCGCTGGTTCGCCTGAATCGCATCGGTAACGGA |
